# Aligning Surgical Practice to an Evidence Base – Past, Present and Future?

**DOI:** 10.5704/MOJ.2203.001

**Published:** 2022-03

**Authors:** S Nayagam

**Affiliations:** Liverpool, United Kingdom

We are experiencing an evolutionary phase in the teaching of orthopaedic surgery. The model of apprenticeship, which has been the foundation for most professions where both knowledge and technical skills need to be taught, continues still. Within this model, the trainer imparts not just the surgical skills of the operating room but the decision-making algorithms that have been formulated through learning, experience, reflection and self-critique. The apprentice learns by emulation and through a cycle of observation – recall – practise under the tutelage of experts. This expertbased training is entrenched in surgery.

Medicine and surgery are, however, sciences and embedded within is the scientific method – the idea leading to a question which is tested through experiment and proof^1^. The 1980’s saw a new wave in medicine. David Sackett – amongst others – encouraged a re-look of how we make decisions and challenged the established norms and prevalent wisdom in medical practice. His personal journey to this end is worth a read^2^. There was a push to appraise the quality of the published literature and to adopt the conclusions only if well-founded by the science within. There was emphasis on good scientific rigour and, for clinical studies, ways to amalgamate the best into summary analyses. This was a departure from the primacy given to the ‘wisdom’ of experts and, to some, a challenge to expertbased medicine^3,4^.

The subsequent four decades have seen evidence-based medicine (EBM) established as a cornerstone of modern medical and surgical practice. In my role as a trainer and teacher, this has been a welcome change. However, evidence-based medicine may have become so pervasive that I sense a danger of the same problems that emerged with expert-based medicine happening again. What do I mean by this? Firstly, expert-based medicine has been subject to influence from the industry. This too is happening in EBM as major trials often have sponsors from the industry^5^. Secondly, in the era of expert-based medicine, clinical practice and decision-making algorithms were referenced to experts, often with little questioning of the rationale, science or proof behind.

Today the same clinical practice and decisions are referenced to published evidence. Whilst this is, in a scientific sense, more robust than the previous era based on experts, much depends on the quality of evidence referenced. Editors and reviewers of the journals sift the submitted material and challenge methodologies, analyses and inferences made before publication. Here in lies a quality-dependent process. Whilst it is true that the impact factor of journals may lend some weight and gravitas to the published evidence, there is a problem in that those journals – especially in orthopaedic surgery – tend to favour only the highest level of evidence and these studies, in turn, often cover a similar area most of the time. The subspecialist areas of orthopaedic surgery are not always represented and this is partly because randomised controlled trials are either difficult to design or fund here. As such, studies with lesser levels of evidence are more common from these subspecialties and often gain publication in smaller journals. So there is variation in ‘qualities’ of the levels of published evidence and quoting evidence without the ability to assess the strength of that evidence independently is the same danger that occurred when practice was expert-based; quoting one expert without the ability to question or understand the rationale or science behind it.

This problem has been compounded by another change in the 21st century - the digital information age. Access to published material in medicine and surgery has never been easier. With all the evidence available through a few taps and swipes of fingers on phones, any clinician can pick and choose a published paper to support a particular point of view. Add to this is the flood of narrative and systematic reviews as well as meta-analyses, many of which are based on historical studies, some poorly done, others serving vested interests, and many leading to a conclusion that “more research is needed”. Are we really better off today?

I believe the answer to this question is a qualified “YES”. The reason it is qualified is because we have yet to teach the skill of assessing the strength of published evidence. This skill is about understanding uncertainty. Good science and good surgery are related to the science of probability. The ability to be explicit about the uncertainty over certain decisions or interventions is at its core. And so what must lie at the heart of training the future orthopaedic surgeon is education on uncertainty and probability that is relevant to clinical work. The surgeon needs to be familiar with the methodologies behind clinical studies as well as statistical entities like odds ratios, likelihood ratios, relative risks, and forest plots. How can one judge the quality of evidence (and not just the level of evidence it is supposed to represent) unless one is educated in understanding the structure of the experiment that was designed to find the answer? It is this skill and ability that will give future surgeons the ability to sort the “wheat from the chaff” in the mountain of published literature^6^. And this will, in turn, allow the surgeon to make independent assessments of what he or she reads, instead of just relying on the designated level of evidence as declared by the publishing journal.

There is an increasing appreciation that the delineation between the tiers in the pyramid of levels of evidence should be blurred^7^. The top positions held by meta-analyses and randomised controlled trials – a scenario fostered by the EBM movement – have seen their worth being questioned as a consequence of industry involvement^5,8,9^. Additionally, there are circumstances where the quality of evidence presented in one tier (for instance Level 3) can be better than the adjacent one above (Level 2) Similarly, some tiers have poorer evidence than ones from the adjacent level below. This blurring of the boundaries is a welcome perspective as it breaks down what has been a very rigid hierarchy. Furthermore, the gold standard experiment in clinical medicine – the randomised controlled trial (RCT) – has also been subject to scrutiny^10^. In surgery, the pragmatic RCT has been touted to be surgery’s answer to the double blind RCT in medicine, is an attempt to bring the ‘real world’ into the experiment. It aims to use the typical group of patients for whom the treatment is meant and for these patients to be treated by a range of surgeons who would normally encounter such patients^11^. This contrasts against an experiment in a single centre seeing a much smaller subset of patients and treated by a smaller group of surgeons for which the conclusions reached may not apply to other centres with different circumstances. At first sight, this seems a good approach. However, there can be procedures in surgery which possess longer and more difficult learning curves which may, if subject to a pragmatic design of RCT, show to produce no difference in outcomes over standard methods.

This danger of the pragmatic RCT was raised by Simpson *et al* citing the possibility of certain techniques being effective under better circumstances; in short, there are some procedures which require surgeons to attain a level of ability beyond the average level of the majority and may well prove effective after this level of skill had been attained^12^. Such circumstances depict a situation where an expert surgeon operating in optimum conditions may achieve different results. In view of this possibility, a recommendation was made for new procedures to be trialled first under such circumstances (by experts in optimum scenarios) before being subject to pragmatic RCTs. Simply, expert-based surgery is not dead.

David Sackett was a strong proponent of an evidence-based practice but what is often forgotten is the emphasis he gave to including clinician knowledge, experience, skill, patient values and their preferences together with the best available evidence for the decision-making process^13^. Expertise can influence decisions; the different levels of clinician knowledge, experience and skill is just another description for expertise. So for each decision we make in clinical care, there are multiple influencing variables. Relying on the results of a RCT or a meta-analysis may not suffice. We need to move a little beyond and adopt a system where all the influencing variables are accounted for. It is probable that some experts were achieving this in the past through the amalgam of knowledge, experience and skill and this may have been borne out through a high number of positive outcomes for their patients, whether as assessed clinically or through patient-reported measures. But is there a way to enable this ability to be taught or even computed? There is some hope that the system of Bayesian networks as used in medicine and surgery may provide an answer^14,15^. These systems will enable the ability to compute the probability of an outcome where the best available evidence is added to expert knowledge, patient influences, causal factors and even the surgeon’s past results. These probabilities can be computed for past data and updated for new and changing data on these variables with time.

It has taken a generation for evidence-based practice to become entrenched in modern clinical life. It brought with it – for orthopaedic surgeons at least – new terminology, new concepts and demands for a better understanding of the structure and quality of clinical experiments. It may take another generation for the blend between expertise, patient characteristics and best available evidence to become a default process in clinical work. This return to appreciating the value of expertise in clinical care is not a case of back to expert-based medicine but a real step forward in as much as modern computing abilities and artificial intelligence will allow us to simplify that collation of information for making individualised decisions for individuals, and not adopt a “one size fits all” approach in treatment.
